# Magnesium supplementation and preeclampsia in low-income pregnant women – a randomized double-blind clinical trial

**DOI:** 10.1186/s12884-020-02877-0

**Published:** 2020-04-09

**Authors:** Carla Adriane Leal de Araújo, Larissa de Sousa Oliveira, Isabela Melo Buarque de Gusmão, Angélica Guimarães, Moranna Ribeiro, João Guilherme Bezerra Alves

**Affiliations:** 1 Department of Pediatrics, Instituto de Medicina Integral Prof. Fernando Figueira (IMIP), Rua dos Coelhos, 300, Boa Vista, Recife, Pernambuco CEP: 50070-550 Brazil; 2 Mother and Child Health, Faculdade Pernambucana de Saúde (FPS), Av. Mal. Mascarenhas de Morais, 4861, Imbiribeira, Recife, Pernambuco CEP: 51150-000 Brazil; 3 Department of Pediatrics, Hospital Dom Malan, R Joaquim Nabuco, S/N, Centro, Petrolina, Pernambuco CEP: 56304-900 Brazil

**Keywords:** Pregnancy, Preeclampsia, Oral magnesium

## Abstract

**Background:**

Preeclampsia is the major cause of maternal morbidity and mortality in developing countries. Magnesium sulfate is considered first-line therapy against eclampsia and magnesium deficiency in pregnancy has been associated with unfavourable perinatal outcomes. However there are doubts if magnesium supplementation during pregnancy can previne preeclampsia especially in population with high nutritional risk. This trial aims to verify the effect of oral magnesium supplmentation on preeclampsia incidence in low income pregnant women.

**Methods:**

This randomized, double-blind, placebo-controlled trial investigated the effect of oral magnesium citrate supplementation for preeclampsia in low-income Brazilian pregnant women, i.e. annual per capita income of US$ 1025 or less. Participants were admitted to the study with gestational age between 12 and 20 weeks. Magnesium serum level was measured pre-randomization and participants with hypermagnesemia were excluded. After randomizationg participants received magnesium citrate capsule (300 mg magnesium citrate) or a daily placebo capsule, until delivery. Intent-to-treat analysis was performed.

**Results:**

A total of 416 pregnant women were screened and 318 enrolled according to the inclusion criteria; 159 for each arm. Twenty-eight pregnant women were lost to follow-up. 55/290 (18.9%) of pregnant women developed preeclampsia; 26/143 (18.1%) in magnesium group and 29/147 (19.7%) in the control group; OR 0.90 (CI 95% 0.48–1.69), *p* = 0.747. No cases of eclampsia were registered.

**Conclusion:**

Oral magnesium supplementation did not reduce preeclampsia incidence in low-income and low-risk pregnant women.

**Trial registration:**

Registered at ClinicalTrials.gov (Identifier NCT02032186), December 19, 2013.

## Background

Preeclampsia is a pregnancy-specific disorder that afects 2 to 8% of pregnancies and is responsible for the death of 63,000 women worldwide every year [1, 2]. Around 25% of these deaths occur in Latin America [3]. Eclampsia is the major cause of maternal morbidity and mortality, especially in undeveloped and developing countries and represents one of the principal reasons for admission to intensive care units [2, 4].

.The cause of pre-eclampsia is not known. There are some evidence about gene variants involved [5]. However the different incidence rates among developed and undeveloped countries suggests a key role of environment factors. Nutritional factors seem to play an important role. Among nutrients, vitamin C and E, L-arginine, calcium and magnesium have been used in preeclampsia treatment as non-pharmalogical intervention [6–11]. A systematic review concluded that a higher total energy and lower magnesium and calcium intake measured during pregnancy were associated with hypertension related to pregnancy [12]. However this review was based on a limited number of studies.

Magnesium is present in grains, green vegetables and seeds but insufficient magnesium intake is common, especially in low-income regions. It is recommended that women consume 280 mg of magnesium per day, increasing in pregnancy [13]. Magnesium deficiency in pregnancy has been associated with unfavourable perinatal outcomes [14]. In 1984 Conradt reported that oral magnesium supplementation during pregnancy was associated with a reduced risk of pre-eclampsia [15]. Li & Tian showed that magnesium gluconate (3 g/day) may efficiently prevent hypertension induced by pregnany in high risk women [16]. D’Almeida et al. studied 50 pregnant women with magnesium oxide and this group had statistically fewer participants who developed pre-eclampsia compared to placebo group [9]. However, a randomized controlled trial found no difference in preeclampsia incidence between 185 pregnant women with Magnesium aspartate and 189 with placebo [17]. Based on all this we developed a large well-designed double-blind randomized trial to verify the effect of oral magnesium supplmentation on preeclampsia incidence in low income pregnant women.

## Methods

### Study design

This randomized, double-blind, placebo-controlled trial investigated the effect of oral magnesium citrate supplementation for preeclampsia in pregnant women. This randomized clinical trial check the hypothesis that oral magnesium supplementation may decrease preeclampsia incidence. The study lasted around 20 weeks and was carried out between November 2016 and January 2018. This trial was conducted in accordance with the most recent CONSORT statement http://www.consort-statement.org. The trial protocol was registered at ClinicalTrials.gov (Identifier NCT02032186). This study is part of the Brazil MAGnesium trial [18], which aim was to determine whether there is a reduction in the composite perinatal outcome – preterm birth, still birth, neonatal death or small for gestationan age – following the administration of oral magnesium citrate 300 mg. Similar methods were used in a recently published study also part of the Brazil MAGnesium trial [19].

### Setting and participants

The study was conducted at Instituto de Medicina Integral Prof. Fernando Figueira (IMIP), Recife, Brazil. The number of infants born each year in IMIP has varied between 5500 and 6500. Pregnant women who attended the low-risk antenatal care clinic were invited to participate in the study. Inclusion criteria were pregnant women with low-income i.e. annual per capita income of US$ 1025 or less, according to the World Bank [20], aging between 18 and 45 years, gestational age between 12 and 20 weeks (based on the last menstrual period among women with a regular menstrual cycle or by first-trimester pregnancy dating ultrasound), a single gestation and currently residents of the city of Recife.

Exclusion criteria were chronic hypertension (systolic blood pressure ≥ 140 mmHg and/or diastolic blood pressure ≥ 90 mmHg) or previous preeclampsia, mental or neurologic disease, uncontrolled known hyperthyroidism, any type of known active parathyroid disease, chronic diarrheal disease, serum creatinine concentration > 1.1 mg/dL, magnesium serum concentration at baseline > 2.6 mg/dL. Before starting magnesium or placebo, serum creatinine and magnesium levels were measured.

### Randomization and intervention

Randomization was conducted in a 1:1 ratio. A table of random numbers to assign participants was prepared by a researcher who did not participate in the data collection.. These numbers were generated using the Random Allocation Software 2.0. Allocation concealment was guaranteed by using sealed and opaque envelopes. The randomization code was released only after participants completed all baseline measurements.

Participants received 300 mg of magnesium citrate capsule or a daily placebo capsule, both identical in colour and shape. All the capsules were manufactured by IMIP’s Department of Phamacology and packages were supplied with sequential numbers. Code break envelopes were supplied by the Department of Pharmacology but not available for the investigaton team. Adverse events, compliance and clinical intercurrences were checked by the research team during the monthly or biweekly routine prenatal visit. Intervention compliance was defined as the ingestion of at least 80% of the prescribed dose.

Participants were discontinued from study in case of symptons or clinical signals due to intake of the capsules or the cancellation of prenatal care at IMIP. Pregnat women who had not delivered at IMIP were also excluded.

### Outcomes

The primary outcome was the presence of preeclampsia (hypertension and proteinuria), presenting after 20 weeks of gestation. Blood pressure was measured by a trained research using a mercury sphygmomanometer and auscultating the Korotkoff sounds with the pregnant women in the sitting position and using an appropriate cuff size. Pregnancy arterial hypertension was defined as systolic blood pressure ≥ 140 mmHg or diastolic blood pressure ≥ 90 mmHg, based on the mean of the two measurements obtained with a 5-min interval between them. Proteinuria was determined in 24 h urine collections. It was considered > 300 mg/24 h or in an isolated sample of urine with proteinuria/creatininuria ratio ≥ 0.3. These measures were performed routinely during all the prenatal care. The diagnosis of preeclampsia was also considered in the absence of proteinuria, based on the presence of hypertension associated with headache, visual turbidity, abdominal pain or altered laboratory tests, thrombocytopenia < 100,000/mm3, hepatic enzyme elevation (double the basal), renal impairment (> 1.1 mg/dL or double the baseline), or pulmonary edema and visual or brain disorders such as headache, scotomas, or convulsions. Secondary outcomes were eclampsia and oral magnesium side effects.

### Ethical considerations

All participants were clarified about the data confidentiality and their capacity to withdraw from the study. The study coordinator (JGA) affirms and upholds the principle of the participant’s right to privacy; anonymity of the pregnant women shall be guaranteed when presenting the data in scientific journals or scientific metings; individual subject clinical information obtained as a result of this research is considered confidential. All participants provided written informed consent. The study was approved by the Ethical Committee on Research of Instituto de Medicina Integral Prof. Fernando Figueira (document number4033–14, CAAE 27026114.4.000.5201), and was registered in the ClinicaTrials.gov (NCT 02032186).

#### Data analysis

The sample size calculation was based assuming a 60% proportional reduction in pre-eclampsia in magnesium group compared with placebo. A power of 0.8 and an alpha of 0.05 and a 1:1 allocation ratio were adopted. We assumed a prevalence f preeclampsia in the placebo group of 19% (on the basis of pilot data from IMIP). With adjustments for a withdrawal rate of 20%, a minimum of 150 women in each group were required. Stata version 12.1 was used for statistical analysis. Chi-squared test for categorical variables and independent *t*-test for continuous variables were used when appropriate. The effect of the intervention on outcomes were reported as adjusted odds ratio with 95% confidence interval. A *p* value < 0.05 was interpreted as statistically significant. Intent-to-treat analysis was conducted.

## Results

**Fig. 1:**
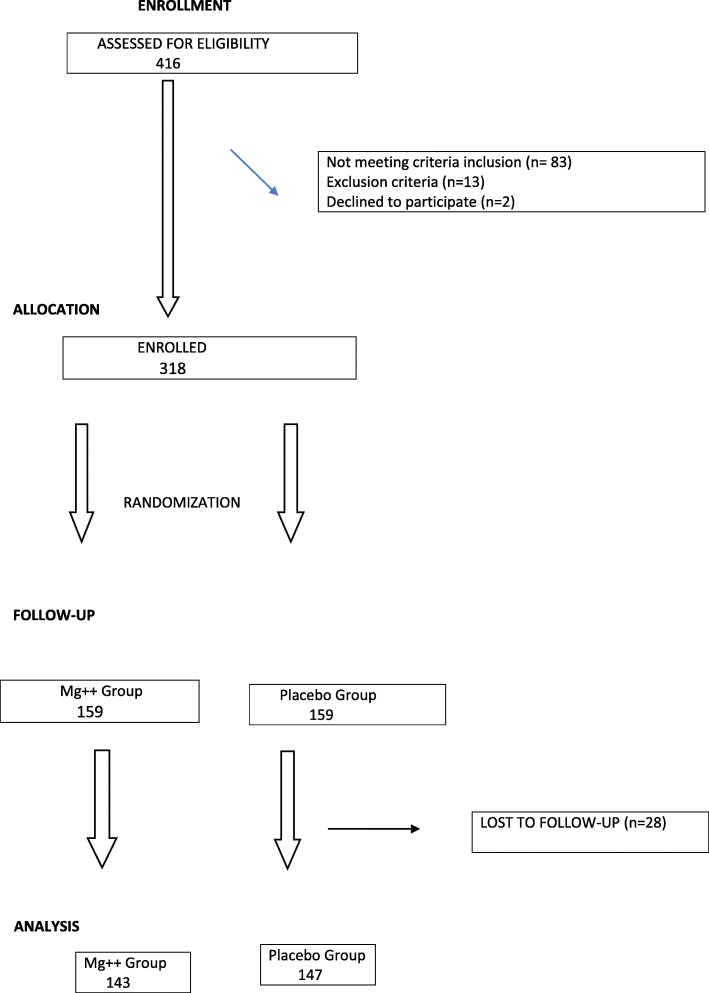
Consort flowchart: Oral magnesium supplementation and preeclampsia

A total of 416 pregnant women were screened and 318 enrolled according to the inclusion criteria; 159 pregnant women were assigned to the Mg++ group (300 mg per day) and 159 were assigned to the placebo (Fig. 1). Twenty-eight pregnant women were lost to follow-up (16 in the magnesium group and 12 in the control group) but 318 participants were included in the intention-to-treat analysis.

The groups showed no significant diferences regarding age, employment, years of study, marital status, per capita income, parity, body mass index, gestational age at admission, and serum magnesium level (Table 1). As there was no difference between the two groups, magnesium and placebo, no regression analysis was performed.

**Table 1:** Some baseline characteristics of pregnant women studied

Characteristic	Magnesium Group *N* = 159 Mean ± SD (%)	Placebo Group *N* = 159 Mean ± SD (%)	*p* value
Age (years)	27.1 ± 5.3	27.5 ± 6.0	0.513^b^
Employed (%)	107 (67.2)	105 (66.0)	0.194^a^
Schooling (years)	4.8 ± 1.0	4.9 ± 1.1	0.341^b^
Marital status - Married (%)	78 (57.4)	72 (47.0)	0.111^a^
Monthly per capita income (US $)	69.80 ± 21.30	72.30 ± 17.50	0.487^b^
Primigesta (%)	90 (56.6)	87 (54.7)	0.220^a^
BMI (Kg/m^2^)	27.0 ± 5.9	26.7 ± 5.3	0.530^b^
Gestation age at admission (weeks)	15.0 ± 3.3	15.6 ± 3.5	0.086^b^
Magnesium (mg/dL)	1.82 ± 0.15	1.81 ± 0.15	0.522^b^
Hypomagnesemia (≤ 1.8 mg/dL)	76 (47.9)	83 (52.1)	0.432^a^

^a^ Qui-square test

^b^ t-studet test

It was observed that 55/290 (18.9%) of pregnant women developed preeclampsia; 26/143 (18.1%) in magnesium group and 29/147 (19.7%) in the control group; *p* = 0.737. All pregnant women with preeclampsia had proteinuria. Symptoms associated with preeclampsia are shown at Table 2. One patient of placebo group had thrombocytopenia. No patients had hepatic enzyme elevation, renal impairment or pulmonary edema. No renal or liver disfunctions were observed.

**Table 2:** Outcome of oral magnesium supplemented group versus placebo group

	Magnesium Group	Control Group	Odds Ratio (95% CI)	p value
Preeclampsia	26	29	0.90 (0.48–1.69)	0.747
Headache	11	14	0.78 (0.35–1.76)	0.566
Visual turbidity	10	7	1.49 (0.56–3.90)	0.427
Abdominal pain	3	5	0.60 (0.15–2.33)	0.491

No cases of eclampsia were registered. Three pregnant women showed gastrointestinal side effects (nauseas and diarrhoea); 1 in magnesium group and 2 in placebo group.

## Discussion

We observed no significant difference in preeclampsia incidence rate between the magnesium supplemented and control groups. Only few studies have been conducted to assess the effect of oral magnesium supplementation to prevent preeclampsia. Our result is in agreement with a recent meta-analysis that identified a nonsignificant correlation between reduced magnesium intakes and hypertensive disorders of pregnancy [12]. Our finding is also similar with another double-blind clinical trial that investigated preeclampsia incidence in a high risk population of 374 pregnant women and identified preeclampsia in 17.3% of oral magnesium supplemented group and 18.5% in placebo group and this difference was not significant [17]. In contrast, Dalmeida et al. investigated preecalmpsia prevention in 150 pregnant women divided in three groups of 50 women who received placebo, magnesium or a mixture of primrose and fish oil; preeclampsia incidence was significantly reduced in magnesium group [9]. However this study has a limitation of a small sample size. We studied low risk pregnant women and interestingly, Bullarbo et al. [21] found that only pregnant women with risk factors for developing hypertension disorders could benefit from extra magnesium intake.

A systematic review found only 3 trial and all were of a low to moderate quality overall [8] and no significant difference in pre-eclampsia rate was observed between the magnesium supplemented and control groups (RR 0.87; 95% CI 0.58 to 1.32; three trials, 1042 women). Two trials (Angola 1992 and China 1997) that showed benefit for the outcome pregnancy-induced hypertension with magnesium supplementation, were of small sample sizes, respectively 150 and 102 participants, and judged to be of a lower quality than the South Africa 2007 trial, of 4476 women, which did not show a difference between groups [9, 16, 22].

Our study showed a high preeclampsia incidence (18.9%). Our region has a high preeclampsia prevalence as comparing to other regions. A previous study in our setting determined an incidence rate of 19.3% of hypertensive disorders of pregnancy [23]. This high incidence could be explained by magnesium deficit but our study with oral magnesium supplementation did not support this hypothesis. A recent study in five different centres in Brazil with 1165 nulliparous healthy pregnant women, found a 7.5% incidence of preeclampsia [24]. This study included centres with different socio-economics conditions.

All participants in our study had a low-income and magnesium serum level was in the minimum limit of normality (1.8 mg/dl) and around half of participants had hypomagnesemia. This seems to indicate that our studied population was at risk of suffering malnutrition and our results can not be generalized. However we measured serum magnesium levels, which may underestimate the prevalence of magnesium deficiency as most magnesium exists intracellularly. Furthermore, according to Ismail et al. [25], normomagnesemia does not exclude magnesium deficiency because only 1% of all magnesium is measurable in the blood, and blood levels decrease only when the deficiency is very serious. The 300 mg daily dose of magnesium citrate used herein approximated that recommended in pregnancy [26]. Although magnesium deficiency has been implicated with an increased risk for adverse perinatal outcomes, there is not enough high-quality evidence to show that dietary magnesium supplementation during pregnancy is beneficial [8].

Our study has strengths and limitations. We conducted a double-blind randomized clinical trial following the CONSORT guidelines. The intervention was carefully planned and carried out and began around the 15th gestational week. We believe all of this has given our finds more reliability. As a limitation, the sample size calculated could detect only a large effect and we did not determine the serum magnesium level after intervention. However the magnesium intake by participants was accomplished. Besides, we studied only low-income pregnant women and our results can not be generalized.

## Conclusions

Oral magnesium supplementation during pregnancy seems to be safe, feasible and inexpensive but is yet not proven to be effective in preventing preeclampsia among low-income and low-risk pregnant women. Interestingly, a great number of studied pregnant women had hypomagnesemia however, oral magnesium supplementation seems not to have contributed to prevent preeclampsia in these women. Further studies are needed.

## Data Availability

The datasets used and/or analysed during the current study are available from the corresponding author on reasonable request.

## Abbreviation

IMIP: Instituto de Medicina Integral Prof. Fernando figueira
